# Body Perception in Newborns

**DOI:** 10.1016/j.cub.2013.10.017

**Published:** 2013-12-02

**Authors:** Maria Laura Filippetti, Mark H. Johnson, Sarah Lloyd-Fox, Danica Dragovic, Teresa Farroni

**Affiliations:** 1 Centre for Brain and Cognitive Development, Birkbeck College, University of London, London WC1E 7HX, UK; 2 Department of Pediatric Unit, Hospital of Monfalcone, Gorizia 34074, Italy; 3 Dipartimento di Psicologia dello Sviluppo e della Socializzazione, University of Padua, Padua 35131, Italy

## Abstract

Body ownership and awareness has recently become an active topic of research in adults using paradigms such as the “rubber hand illusion” and “enfacement” [1–11]. These studies show that visual, tactile, postural, and anatomical information all contribute to the sense of body ownership in adults [12]. While some hypothesize body perception from birth [13], others have speculated on the importance of postnatal experience [14, 15]. Through studying body perception in newborns, we can directly investigate the factors involved prior to significant postnatal experience. To address this issue, we measured the looking behavior of newborns presented with visual-tactile synchronous and asynchronous cues, under conditions in which the visual information was either an upright (body-related stimulus; experiment 1) or inverted (non-body-related stimulus; experiment 2) infant face. We found that newborns preferred to look at the synchronous condition compared to the asynchronous condition, but only when the visual stimulus was body related. These results are in line with findings from adults and demonstrate that human newborns detect intersensory synchrony when related to their own bodies, consistent with the basic processes underlying body perception being present at birth.

## Highlights

• Human newborns are able to detect synchronous intersensory stimulation
• Newborns prefer to look at body-related, synchronous visual-tactile stimuli
• Key processes underlying body perception are present at birth

## Results and Discussion

**Figure 1:**
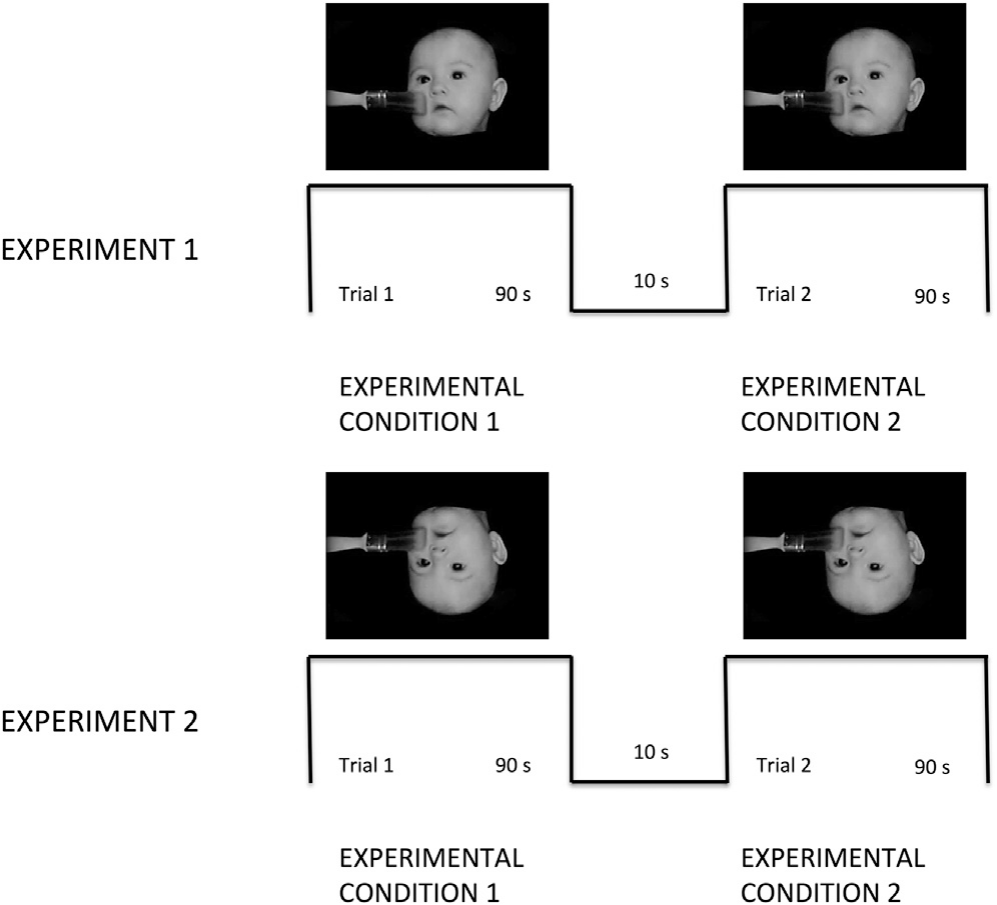
Experimental Design An example of the video stimuli used in the study. Experiment 1: visual-tactile synchronous compared to visual-tactile asynchronous stimulation using body-related information (a dynamic upright infant face). Experiment 2: visual-tactile synchronous compared to visual-tactile asynchronous stimulation using non-body-related information (a dynamic inverted infant face). In the synchronous condition, the newborn was touched on the cheek with a paintbrush on the specularly congruent location, and the strokes perfectly matched (e.g., temporally and spatially) the brush stroke on the infant’s corresponding cheek displayed on the screen. In the asynchronous condition, the newborn was again touched on the cheek, but the tactile stimulation was delayed with regard to the brush stroke displayed on the screen by 5 s. An experimenter who stood behind the infant to prevent them from being distracted delivered stroking manually. Each stroke lasted approximately 1 s and started on the middle of cheek and ended at the beginning of the ear. In experiment 2, the newborns were presented with the same videos as in experiment 1, but this time the image was inverted using video-editing software (Adobe Premiere Pro CS6) to rotate the video by 180°. In order to counterbalance the side of the stroke and still keep constant the spatial congruency between touched and observed cheek, we used mirrored versions of the videos in both experiments.

Studies investigating body ownership and awareness in adults have highlighted the importance of the temporal synchrony and spatial congruency of sensory stimuli [12], in addition to body morphology and anatomical posture [4, 5, 10, 16]. These studies show that body-related cues, here defined as information attributable to the current position of one’s own body, are fundamental for body perception. Despite several studies on infants that have shown the important role of proprioception (where movements are self-produced) and multisensory integration in the development of body awareness during infancy [17–20], to our knowledge, only one study has investigated the role of body-related synchrony detection during infancy solely based on afferent information [21]. However, it remains unknown whether these factors play a role from birth or whether the detection of body-related intermodal synchrony develops gradually with experience throughout infancy. We addressed this issue and hypothesized that, in the absence of any visual feedback from self-generated movements, newborns would show a preference (as measured by longer looking) to a synchronous visual-tactile condition when viewing a video of an upright infant face as compared to an asynchronous, temporally delayed condition (experiment 1). Furthermore, we predicted that this increased attention to intersensory synchrony would not be present in the context of a visual stimulus that did not resemble themselves (the same infant face video, but inverted; experiment 2). By inverting the visual stimulus, we reduced the likelihood that the newborns would relate it to their own bodies [12, 18, 21]. In other words, rather than a general preference for synchrony of observed and felt actions, newborns would prefer this perfect matching only in the context of stimuli that are related to their own bodies (see an example of the experimental paradigm in Figure 1).

**Figure 2:**
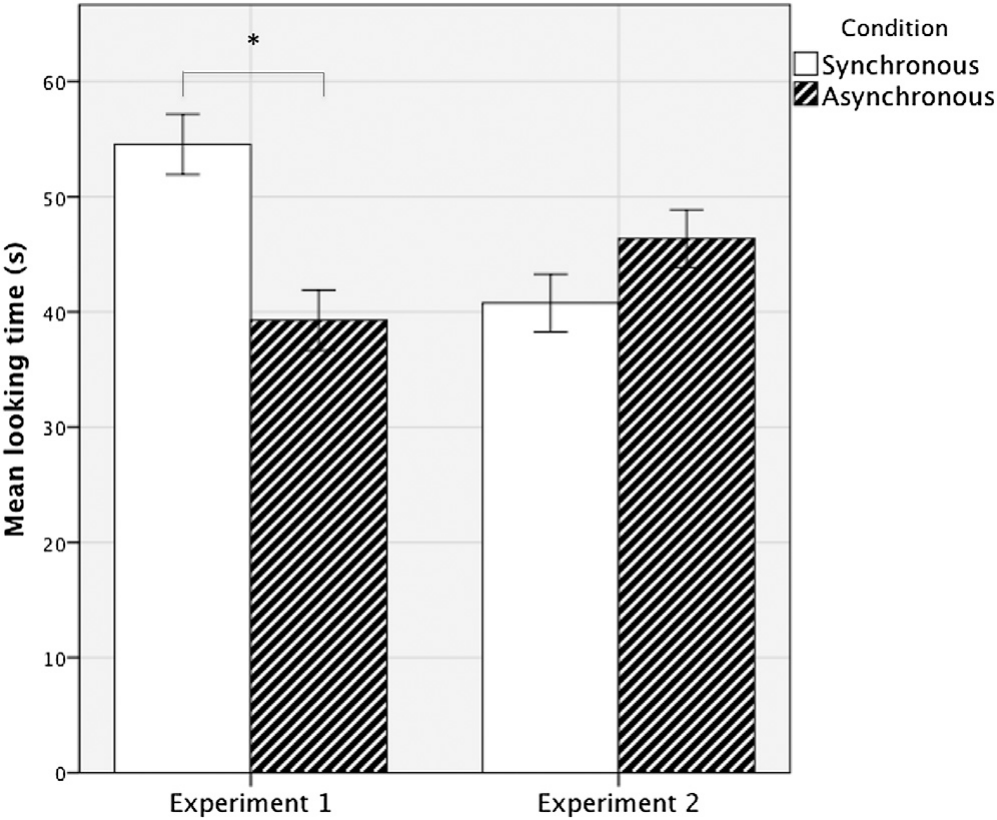
Looking Time Results Mean and SE of looking times to the synchronous and asynchronous stimuli in experiments 1 and 2. Only in experiment 1 was an effect of synchrony observed.

The data from the two experiments were analyzed with a two-way mixed ANOVA, with visual-tactile stimulation (synchronous versus asynchronous) as a within-subject factor and type of video (upright versus inverted) as a between-subject factor. The analysis showed that while there were no significant main effects of inversion [F(1,38) = 0.48, p = 0.49] or synchrony [F(1,38) = 1.79, p = 0.19], the predicted interaction effect was significant [F(1,38) = 8.29, p = 0.007, *r* = 0.80], indicating that the looking time during the synchronous and asynchronous conditions differed according to whether the video was upright or inverted (see Figure 2). To investigate this interaction further, we performed two separate paired-sample t tests. The results of experiment 1 showed that there was a significant difference between the synchronous (mean = 54.54, SE = 3.66) and asynchronous (mean = 39.28, SE = 5.04) conditions [*t*(19) = 2.92, p = 0.009]. The results of experiment 2, in which newborns were presented with synchronous and asynchronous visual-tactile stimulation while they watched the inverted face video, showed no significant difference between the two conditions [synchronous condition, mean = 40.77, SE = 4.02; asynchronous condition, M = 46.36, SE = 4.18; *t*(19) = −1.12, p = 0.30; see Table 1 for additional analyses]. Several studies on human newborns (for a review, see [22]) have shown that they respond differentially to viewing upright, as compared to inverted, face-like stimuli. Results of the present study confirm the importance of the upright face for the detection of synchronous intersensory information. There was no effect of order of presentation of the conditions in either experiment [experiment 1, F(1,18) = 1.62, p = 0.22; experiment 2, F(1,18) = 2.52, p = 0.13].

The results of experiment 1 could be interpreted as a preference for redundant sensory information and are in line with previous studies demonstrating that newborns are able to integrate intersensory stimulation [23, 24]. Our finding is also consistent with the intersensory redundancy hypothesis (IRH), which highlights the importance of amodal information, such as temporal synchrony, for early perceptual development and learning [25, 26]. However, our results go beyond this conclusion as we show a preference for visual-tactile temporal synchrony only when the visual stimulus presented is relevant to the infant’s own body. Since we did not use live video of the watching infant herself, we are able to rule out the contingent visual feedback from the viewing infant’s own self-performed actions. These findings are in accord with previous studies in older infants and adults, in which it has been shown that the illusion of owning a specific body part occurs only when the fake limb matches the physical and morphological features of the real one [10, 18, 21].

To our knowledge, only one other study has investigated the role of body-related synchrony detection during infancy in the absence of any efferent information [21]. These authors demonstrated that, when presented with a video display of life-like baby doll legs, infants aged 7 and 10 months discriminate between contingent and noncontingent visual-tactile information. Importantly, because the infants were watching baby doll legs and could not control the visual feedback of their movements, they demonstrated the ability to discriminate contingency independently from movements of their own bodies [21]. Furthermore, the authors observed that this preference disappears when 10-month-old infants are presented with nonbody objects (i.e., wooden blocks), showing that morphological similarities between the stimulus and their own bodies are important in the detection of visual-tactile contingency at this age [21]. In the present experiments, we tested the role of body-related stimuli with newborns, finding that from the first days of life, infants can detect intersensory synchrony when related to their own bodies. Previous studies on infants showed that 3 s delay is sufficient for 3-month-old infants to differentiate between synchrony and asynchrony [27, 28]. We now provide evidence that newborns can discriminate between synchrony and asynchrony when presented with 5 s delay.

We do not know whether the newborns in our studies attributed the upright dynamic face seen on the screen as directly belonging to their own bodies. However, our findings are in line with research on self-identification in adults [12]. Studies on “enfacement” in adults show that seeing another person’s face being touched synchronously with one’s own face evokes a change in self-face recognition, whereby the other face becomes incorporated to some extent into the representation of one’s own face [9]. In these studies, the “other” becomes included in the mental representation of one’s own face as a consequence of viewing a perfect matching between the seen and felt sensory stimulation in the context of watching the other person’s face. In order to maintain a coherent and updated sense of one’s body, the internal body model and the new, external information provided are compared together, and irrelevant or incongruent information is discarded [29].

To our knowledge, this is the first study to investigate visual-tactile stimulation of faces in infants, solely based on afferent information. Legerstee et al. [30] showed that 5- and 8-month-old infants look longer at video of their own faces compared to those of peers and dolls, but only when it is moving (live video feed). This highlights the importance of self-generated movement by means of a matching between their executed actions and the visual feedback. In the present research, because the matching between seen and felt touch was the only congruent information that the newborns could rely on, we demonstrated that perception of visual-tactile synchrony may be important for differentiating between self and other in the absence of any self-generated movement.

In the current study, we specifically investigated the role of temporal synchrony in multisensory integration. In future work, it will be important to examine the role of bodily location as a constraint on intermodal perception. We know from research with adults that a rubber hand located in an incongruent anatomical and postural position reduces the illusion of owning that hand [12]. It is worth investigating whether a change in the bodily location of the touch compared to that observed on the video would eliminate the preference for visual-tactile temporal synchrony in newborns. This would strengthen the view that bottom-up sensory information and top-down cues related to one’s own body are present at birth and together help the newborn to form a reliable percept of her own body across development. Future work could also examine the extent to which attention to particular visual features is critical for the effect we have observed.

The current findings have important implications for our understanding of the development of body awareness. In accord with the infancy literature on the development of body awareness [15, 17], we propose that intermodal detection of synchrony provides important information allowing infants to differentiate the self from others and to form a coherent representation of their own bodies. Our results provide the first evidence of body-related visual-tactile temporal synchrony detection in newborns.

## Experimental Procedures

### Participants

The study was conducted at the Pediatric Unit of the Hospital of Monfalcone. Forty newborns (20 female; ten in experiment 1 and ten in experiment 2) from 12 to 103 hr of age at time of test took part in the study; 20 additional newborns also participated but were excluded due to fussiness (ten), low intercoder reliability (four), or equipment failure (six). All the newborns that completed the study met the screening criteria of normal delivery, had a birth weight of more than 2,500 g, and had an Apgar score of at least 8 at 5 min. No abnormalities were present at birth.

The 20 newborns that participated in experiment 1 had a mean age of 45.2 hr (SD = 19.8), with a mean gestational age of 39.7 weeks (SD = 1.53). The 20 newborns that participated in experiment 2 had a mean age of 49.8 hr (SD = 26.89), with a mean gestational age of 39.3 weeks (SD = 1.03). The testing took place when the baby was awake and alert, usually during the hour preceding the feeding time. Parents were informed about the procedure and gave their consent to their child’s participation. The local ethics committee approved the study protocol.

### Apparatus and Stimuli

The newborns sat on the experimenter’s lap in a research room within the hospital. The distance between the monitor (size 27 in or 69 cm) and the newborn’s head was approximately 30 cm [31, 32]. The newborn’s eye level was aligned to the center of the screen. A video camera was placed on the top of the screen and filmed the infant’s eyes, allowing the experimenter to monitor his/her eye movements.

The visual stimuli in all the experiments consisted of two identical—previously recorded—videos, one displayed on the left of the screen and one on the right to ensure that newborns’ attention was engaged and to avoid sticky fixation (though it is important to bear in mind that the use of the same face shown side by side during the experimental session may have affected the behavior of the infants, potentially preventing them from relating their faces to one of the two stimuli).

In both experiments, these videos presented a 5-month-old infant face being stroked with a paintbrush every 10 s from a specular point of view, just as the neonate watching the screen would see her face being touched in front of a mirror. Therefore, in both experiments, the viewing newborn’s side of the stroke was always spatially congruent with the side of the face seen stroked on the screen. The two identical faces subtended a visual angle of 18.4° × 21.4° each, and together the two experimental stimuli subtended a visual angle of 18.4° × 58.4°. In all of the stimuli, the pupil was 1 cm in diameter and the pairs of faces were 13.3 cm apart. The stimuli were presented using E-Prime 2.0.10. A prerecorded unfamiliar infant face was chosen because we assumed that newborns from 12 to 103 hr have no experience of their own specular images in a mirror, and so don’t know the invariant features of their own faces.

The two different conditions investigated in each of the two experiments are illustrated in Figure 1.

### Procedure

Once the newborn was seated and fixated upon the center of the screen, the experiment began. One synchronous and one asynchronous trial were presented to each newborn. The order of presentation of the two trials and the cheek on which the infant was stroked were counterbalanced across infants. Each trial lasted 90 s, interleaved with a 10 s rest period (blank screen). Directly before the rest period, a flashing icon was presented to maintain the newborn’s attention.

Each trial was divided into eight segments (10 s each), where a segment started from the first frame in which the paintbrush touched the infant cheek to the first frame in which the next paintbrush appeared on the screen. The first stroke was presented after 3 s of stimulus presentation, followed by 10 s of video before the next stroke was presented, and so on. Within a 90 s trial, there were eight strokes in total. Therefore, each stroke was presented on the screen approximately every 10 s and, depending on the condition, was either synchronous with the touch delivered on the newborn’s face or mismatched and presented with a lag of 5 s relative to the touch. Based on the video recordings, two independent observers coded how long each newborn looked at the monitor (the second observer was naive to the hypothesis) and total looking time was measured for each trial. The analysis was conducted by comparing the looking time values of the two coders. Two interrater reliability analyses were performed: Pearson’s r correlation analysis and Cohen’s kappa reliability analysis. The Pearson’s r correlation was performed on the total sample and was *r* = 0.96 for experiment 1 and *r* = 0.83 for experiment 2. Cohen’s kappa analysis was performed for 20% of the sample of each experiment, revealing scores of k = 0.73 for experiment 1 and = 0.74 for experiment 2.

Despite having chosen a 90 s trial length for each experimental condition, we decided to take into account the effective interest of the infant by applying an offline infant-control procedure, allowing us to measure the actual fixation time. Therefore, when the infant looked away for over 10 s, the remaining section was discarded from the looking time analysis (see Table 1). Furthermore, if the newborn did not see the brush touching the infant’s face, the 10 s segment that included this brush stroke was excluded from further analyses [note that the final results remain the same if looking time during the segments where the newborn did not see the brush is also included in the analyses; *t*(19) = 3.05, p = 0.007 for experiment 1; synchronous condition, mean = 59.30, SE = 3.54; asynchronous condition, mean = 44.29, SE = 4.93; *t*(19) = −0.82, p = 0.42, for experiment 2; synchronous condition, mean = 45.38, SE = 4.25; asynchronous condition, mean = 49.35, SE = 4.01]. This exclusion criterion was applied in order to be confident that the synchronicity of the visual and tactile information was perceived by the newborn. Indeed, we assumed that if the infant did not see the brush on the screen, the integration of the sensory information did not occur.

## Figures and Tables

**Table 1:** Number and Distribution of Trials for which Data Were Discarded Using an Offline Infant-Control Procedure

Experiment	Synchronous				Asynchronous			
	Total Trials	M	SD	Excluded Trials	Total Trials	M	SD	Excluded Trials
Experiment 1 (Upright)	143	6.6	1.7	11	136	5.3	2.6	31
Experiment 2 (Inverted)	136	5.5	2.2	13	140	6.4	2.1	27

In experiment 1, the criterion applied to ten out of 20 infants (five in the synchronous condition and eight in the asynchronous condition). In experiment 2, the criterion applied to 11 out of the 20 newborns tested (eight in the synchronous condition and six in the asynchronous condition). Note that the final results remain the same even if this exclusion criterion is not applied [*t*(19) = 2.27, p = 0.035 for experiment 1; synchronous condition, mean = 57.57, SE = 3.23; asynchronous condition, mean = 49.46, SE = 3.02; *t*(19) = −1.12, p = 0.30 for experiment 2; synchronous condition, mean = 46.81, SE = 3.13; asynchronous condition, mean = 50.99, SE = 3.34].

## References

[1] Apps M.A., Tajadura-Jiménez A., Turley G., Tsakiris M. (2012). The different faces of one’s self: an fMRI study into the recognition of current and past self-facial appearances. Neuroimage.

[2] Botvinick M., Cohen J. (1998). Rubber hands ‘feel’ touch that eyes see. Nature.

[3] Ehrsson H.H., Holmes N.P., Passingham R.E. (2005). Touching a rubber hand: feeling of body ownership is associated with activity in multisensory brain areas. J. Neurosci..

[4] Guterstam A., Petkova V.I., Ehrsson H.H. (2011). The illusion of owning a third arm. PLoS ONE.

[5] Kalckert A., Ehrsson H.H. (2012). Moving a rubber hand that feels like your own: a dissociation of ownership and agency. Front. Hum. Neurosci..

[6] Makin T.R., Holmes N.P., Ehrsson H.H. (2008). On the other hand: dummy hands and peripersonal space. Behav. Brain Res..

[7] Sforza A., Bufalari I., Haggard P., Aglioti S.M. (2010). My face in yours: visuo-tactile facial stimulation influences sense of identity. Soc. Neurosci..

[8] Tajadura-Jiménez A., Grehl S., Tsakiris M. (2012). The other in me: interpersonal multisensory stimulation changes the mental representation of the self. PLoS ONE.

[9] Tajadura-Jiménez A., Longo M.R., Coleman R., Tsakiris M. (2012). The person in the mirror: using the enfacement illusion to investigate the experiential structure of self-identification. Conscious. Cogn..

[10] Tsakiris M., Haggard P. (2005). The rubber hand illusion revisited: visuotactile integration and self-attribution. J. Exp. Psychol. Hum. Percept. Perform..

[11] Tsakiris M. (2008). Looking for myself: current multisensory input alters self-face recognition. PLoS ONE.

[12] Tsakiris M. (2010). My body in the brain: a neurocognitive model of body-ownership. Neuropsychologia.

[13] Neisser U. (1991). Two perceptually given aspects of the self and their development. Dev. Rev..

[14] Rochat P. (2009). Others in Mind: Social Origins of Self-Consciousness.

[15] Striano T., Rochat P. (2000). Perceived self in infancy. Infant Behav. Dev..

[16] Longo M.R., Schüür F., Kammers M.P.M., Tsakiris M., Haggard P. (2009). Self awareness and the body image. Acta Psychol. (Amst.).

[17] Bahrick L.E., Watson J.S. (1985). Detection of intermodal proprioceptive–visual contingency as a potential basis of self-perception in infancy. Dev. Psychol..

[18] Rochat P., Morgan R. (1995). Spatial determinants in the perception of self-produced leg movements in 3-to 5-month-old infants. Dev. Psychol..

[19] Schmuckler M.A. (1996). Visual–proprioceptive intermodal perception in infancy. Infant Behav. Dev..

[20] Watson J.S., Parker S.T., Mitchell R.W., Boccia M.L. (1994). Detection of self: the perfect algorithm. Self-Awareness in Animals and Humans: Developmental Perspectives.

[21] Zmyj N., Jank J., Schütz-Bosbach S., Daum M.M. (2011). Detection of visual-tactile contingency in the first year after birth. Cognition.

[22] Johnson M.H. (2005). Subcortical face processing. Nat. Rev. Neurosci..

[23] Lewkowicz D.J., Ghazanfar A.A. (2009). The emergence of multisensory systems through perceptual narrowing. Trends Cogn. Sci..

[24] Lewkowicz D.J., Leo I., Simion F. (2010). Intersensory perception at birth: newborns match nonhuman primate faces and voices. Infancy.

[25] Bahrick L.E., Lickliter R. (2000). Intersensory redundancy guides attentional selectivity and perceptual learning in infancy. Dev. Psychol..

[26] Bahrick L.E., Lickliter R., Bremner A., Lewkowicz D.J., Spence C. (2012). The role of intersensory redundancy in early perceptual, cognitive, and social development. Multisensory Development.

[27] Gergely G., Watson J., Rochat P. (1999). Early socio-emotional development: contingency perception and the social-biofeedback model. Early Social Cognition.

[28] Zmyj N., Hauf P., Striano T. (2009). Discrimination between real-time and delayed visual feedback of self-performed leg movements in the first year of life. Cogn. Brain Behav..

[29] Tsakiris M., Costantini M., Haggard P. (2008). The role of the right temporo-parietal junction in maintaining a coherent sense of one’s body. Neuropsychologia.

[30] Legerstee M., Anderson D., Schaffer A. (1998). Five- and eight-month-old infants recognize their faces and voices as familiar and social stimuli. Child Dev..

[31] Fantz R.L., Ordy J.M., Udelf M.S. (1962). Maturation of pattern vision in infants during the first six months. J. Comp. Physiol. Psychol..

[32] Slater A., Earle D.C., Morison V., Rose D. (1985). Pattern preferences at birth and their interaction with habituation-induced novelty preferences. J. Exp. Child Psychol..

